# Shale gas geological “sweet spot” parameter prediction method and its application based on convolutional neural network

**DOI:** 10.1038/s41598-022-19711-6

**Published:** 2022-09-13

**Authors:** Zhengye Qin, Tianji Xu

**Affiliations:** 1 School of Resources and Environment, University of Electronic Science and Technology of China, Chengdu, 611731 Sichuan China; 2 Yangtze Delta Region Institute, University of Electronic Science and Technology of China, Huzhou, 313000 Zhejiang China

**Keywords:** Geophysics, Seismology

## Abstract

Parameters such as gas content (GAS), porosity (PHI) and total organic carbon (TOC) are key parameters that reveal the shale gas geological “sweet spot” of reservoirs. However, the lack of a three-dimensional high-precision prediction method is not conducive to large-scale exploration of shale gas. Although the parameter prediction accuracy based on well logging data is relatively high, it is only a single point longitudinal feature. On the basis of prestack inversion of reservoir information such as P-wave velocity and density, high-precision and large-scale “sweet spot” spatial distribution predictions can be realized. Based on the fast growing and widely used deep learning methods, a one-dimensional convolutional neural network (1D-CNN) “sweet spot” parameter prediction method is proposed in this paper. First, intersection analysis is carried out for various well logging information to determine the sensitive parameters of geological “sweet spot”. We propose a new standardized preprocessing method based on the characteristics of the well logging data. Then, a 1D-CNN framework is designed, which can meet the parameter prediction of both depth-domain well logging data and time-domain seismic data. Third, well logging data is used to train a high-precision and robust geological “sweet spot” prediction model. Finally, this method was applied to the WeiRong shale gas field in Sichuan Basin to achieve a high-precision prediction of geological “sweet spots” in the Wufeng–Longmaxi shale reservoir.

## Introduction

Shale gas is a natural gas resource stored in shale reservoirs and exists in natural fractures and pores in free and adsorbed forms^1^. China’s shale gas reserves are abundant, but the geological structure is complex and the reservoirs are deeply buried. In addition, the total organic carbon and gas contents of shale reservoirs change rapidly and have poor predictability, which leads to difficulties in obtaining these parameters by traditional methods^2^. Therefore, it is of great importance to explore new methods for predicting the “sweet spot” parameters of shale reservoirs based on deep learning methods.

The “sweet spot” of shale reservoirs refers to areas or horizons where shale gas is relatively rich, easy to develop, and economically beneficial. The “sweet spot” can be divided into geological “sweet spot” and engineering “sweet spot” at different stages of research^3^. Among the various parameters that reveal the geological “sweet spot”, porosity (PHI) and permeability (PERM) are geophysical parameters, and total organic carbon content (TOC) is a geochemical parameter. Gas content (GAS) is also an important parameter for evaluating shale gas reservoirs. The “sweet spot” area has higher TOC, PHI, PERM and GAS. For engineering, pressure coefficient and brittleness index are important “sweet spot” parameters, which reveal the feasibility of fracturing. The methods to obtain the “sweet spot” parameters of the reservoir include core testing, logging, and seismic prediction. Among them, the test analysis based on core samples has limitations, such as a high sampling cost and long period. The data is usually scarce and discontinuous. The instability of the sample may introduce errors during sampling and analysis^4^. The logging response is complex and is affected by the mineralogy and pore fluid properties of the rock and organic matter^5^. Well logging data can reflect the longitudinal lithology and strata changes of the well location and have the advantages of continuity and high longitudinal resolution compared to core data. According to the sensitive parameters of source rock in logging data, such as acoustic log (AC), density (RHOB), and natural gamma ray (GR), the “sweet spot” parameters of shale reservoirs can be predicted. However, limited by cost and construction conditions, logging data is scarce. Logging data is limited by a “one hole view”, which can only show the characteristics of the “sweet spot” parameter in a small range of longitudinal variations near the borehole^6^. Seismic data is indispensable for extracting the lateral change characteristics of the region. However, parameters, such as TOC and GAS cannot be directly obtained through seismic exploration. Generally, it is predicted based on data, such as transverse and longitudinal wave velocity and density using empirical formulas or regression fitting and other conventional methods^7^. Xu analyzed the effect of nanoscale pore size distribution on shale gas production, and the permeability and porosity are affected by geomechanical effect^8^.

The conventional method predicts “sweet spot” parameters by the linear fitting of elastic parameters, but it has disadvantages, including low prediction accuracy and regional limitations. Tan et al. used natural gamma spectroscopy logging, multiple regression and $\Delta$LogR methods to predict TOC and found that multiple regression and natural gamma spectroscopy logging had a positive effect on shale reservoir TOC prediction in the southern Sichuan Basin^9^. Zhang et al. established a shale linearized petrophysical model and, based on the Bayesian linear inversion framework, predicted the “sweet spot” parameters, such as TOC, PHI and brittle minerals from the elastic parameter inversion volume^10^. Li et al. used support vector machines to achieve TOC prediction^11^. Clustering was used to classify different lithological formations, improve the correlation between logging parameters and TOC content, and then to establish SVR models for different lithological formations to achieve TOC prediction.

Compared with traditional prediction methods and other machine learning methods, neural networks can accomplish complex nonlinear mapping between input features and output results. As the amount of data increases and new methods are continuously proposed, traditional geophysical research can be combined with advanced data science methods^12^. In the analysis of logging and seismic data, due to the heterogeneity of the formation and the complexity of the influencing factors of the parameters, the feature mapping relationship between parameters is complicated. Therefore, neural networks have great potential in the regression prediction of logging and seismic curves^13^. Lu et al. used a back propagation neural network (BP) to predict TOC in the Lunpola Basin and obtained good results^14^. However, the BP neural network has shortcomings including overfitting and poor generalizability. In 1982, Fukushima proposed a self-organizing neural network model for solving pattern recognition problems, in which the concept of convolutional layer and pooling layer was introduced into the neural network^15^. Lecun applied back propagation algorithm to the convolutional neural network (CNN) and got better results^16^. The LeNet-5 proposed in 1998 is a classic and basic CNN model^17^. Since then the theories related to the CNN model and its applications have begun to develop rapidly. The CNN models have stronger feature extraction ability and computing performance. Compared with the BP neural network, the CNN is less likely to fall into local optimal solutions and can preserve the spatial distribution of features, which is more conducive to the prediction of reservoir “sweet spot” parameters.

Therefore, this paper aims to resolve the problems with the lack of prediction methods, the low prediction accuracy and the difficulty in meeting the needs of exploration for the “sweet spot” parameters of shale reservoirs. A one-dimensional convolutional neural network (1D-CNN) is used to establish a high-precision prediction method for the “sweet spot” parameters of the reservoir. Through a standardized preprocessing of logging samples and an optimized design of the 1D-CNN framework, “sweet spot” prediction models are trained based on logging data combined with high-precision P-wave velocity, density and other prestack inversion data to achieve, such as TOC, GAS, PHI, and high-precision intelligent prediction of “sweet spot” parameters. This method is applied to predict the “sweet spot” parameters of the Wufeng–Longmaxi Formation shale reservoir in the Weirong shale gas field in the Sichuan Basin, which provides method support for the optimization of exploration targets and the improvement of single-well production.

## Methodology

To achieve high-precision shale gas prediction, 1D-CNNs are applied to predict “sweet spot” parameters, which are often used for the prediction and classification of one-dimensional data, such as signal sequences^18^. The longitudinal resolution of logging data is much higher than that of seismic data. When using an image convolutional neural network (two-dimensional) or cyclic neural network, it is necessary to ensure the longitudinal consistency of logging and seismic data. The depth-time domain conversion method is used to match logging data and seismic data^19^, which greatly reduces the number of samples, loses feature mapping, and then decreases the prediction accuracy of the model. The 1D-CNN can achieve both depth-domain logging data and time-domain seismic data prediction. Additionally, it also makes full use of logging data feature information.

### One-dimensional convolutional neural network

The input and output of a one-dimensional convolutional neural network (1D-CNN) are in the form of vectors. For geological “sweet spot” parameter prediction, the input of network are “sweet spot” sensitive features, such as longitudinal wave velocity (VP), transverse wave velocity (VS) and density (RHOB). The output is the target parameter (TOC, PHI, GAS). Input features are abstracted as high-level features while passing through the convolutional layers and pooling layers, and by updating the network weights, the feature information in the well logging data can be accurately extracted.

#### One-dimensional convolution and pooling

**Figure 1:**
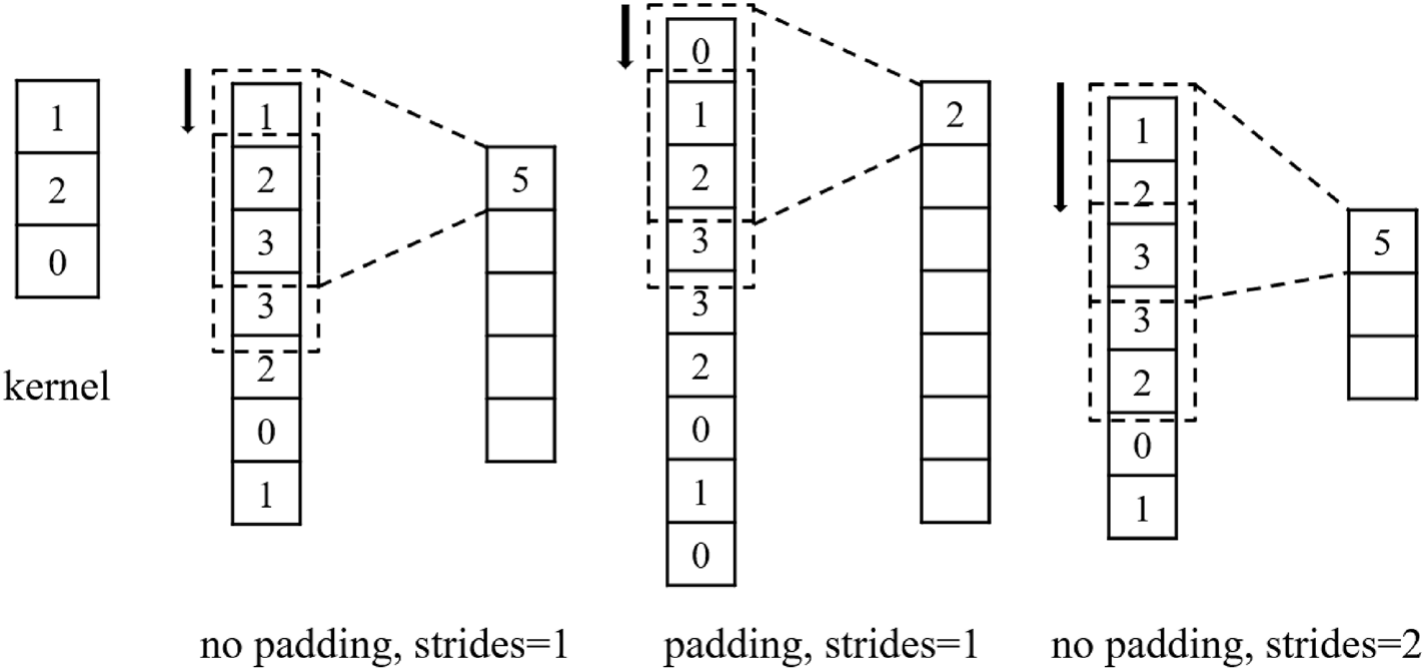
This is a schematic diagram of one-dimensional convolution.

The principle of one-dimensional convolution is shown in Fig. 1. A one-dimensional convolution kernel of length 3 performs convolution on a vector of length 7. The corresponding unit values are multiplied and then accumulated to obtain the convolutional value. Zero padding is added to the beginning and end of the vector and then convolution is performed to make the feature length unchanged after convolution. Appropriately increase the strides of the convolution kernel movement to greatly reduce the feature length while convolution.

The output of the convolutional layer passes through a nonlinear activation function, which enables the model to learn nonlinear features. There are two activation functions used in this method: rectified linear unit (ReLU) and sigmoid function, which can be represented as follows:

1 $$\begin{aligned} f(x)= & {} \max \left\{ {0,x} \right\} \end{aligned}$$

2 $$\begin{aligned} f(x)= & {} \frac{1}{{{e^{ - x}} + 1}} \end{aligned}$$

**Figure 2:**
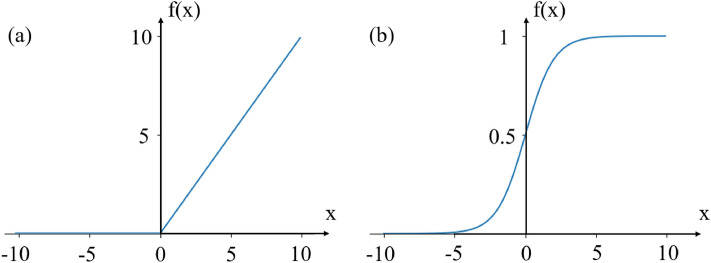
ReLU activation function (**a**) and Sigmoid activation function (**b**).

Figure 2 depicts the graphs of these two activation functions. The sigmoid function has an effect of normalization so that the output range can be controlled between (0, 1), which fits well with the normalized label value. Its advantage is that the function is continuous and smooth so it is easy to derive. However, when the input value is too small or too large, it is easy to cause the gradient disappearance problem, and differentiation operation of sigmoid function is relatively slow. Therefore, the sigmoid function is usually used in the output layer. The curve of the Tanh function is similar to that of the sigmoid function, but the output range is (− 1, 1). The disadvantages of gradient disappearance and slow calculations are not fully resolved.

The ReLU function is characterized by sparse activation and ignores negative values, thereby reducing overfitting. Its gradient is only 0 or 1, which solves the gradient disappearance problem and its derivative calculation is fast. ReLU function has been widely used in CNNs and has a stable performance. Although there are many improved versions of ReLU function, such as ELU function and softplus function, it has not been proven that there must be better results with them. So the simplest and fastest ReLU function is still preferred for networks, and when the model is not working well, more complex activation functions can be considered.

**Figure 3:**
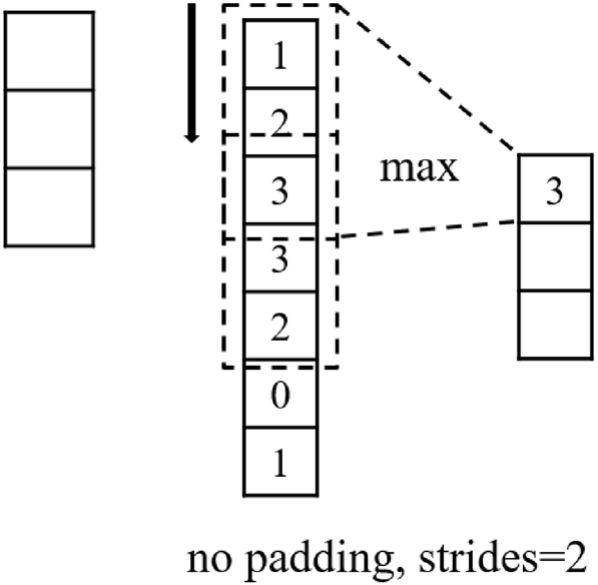
This is a schematic diagram of one-dimensional max-pooling.

In CNNs, the pooling layer can quickly reduce the size of the feature maps and improve the training speed. Commonly used pooling methods include average-pooling and max-pooling. Average-pooling is essentially a convolution operation with equal weights. Max-pooling selects the maximum value in the pooling window as the output, which can enhance nonlinearity. Figure 3 shows a max-pooling window of length 3 to pool a vector of length 7, with a stride of 2, and the output is a vector of length 3.

#### Loss function

Loss function is the error between the output value and the sample value, which serves as the basis for updating the network weight. The input features pass through the hidden layers to obtain the output and then calculate the loss function. This process is called forward propagation. The commonly used loss function for regression tasks is the mean square error (MSE) or the mean absolute value error (MAE), which is defined as follows:

3 $$\begin{aligned} MSE= & {} \frac{1}{n}{\sum \limits _{i = 1}^n {\left( {{y_i} - \widehat{{y_i}}} \right) } ^2} \end{aligned}$$

4 $$\begin{aligned} MAE= & {} \frac{1}{n}\sum \limits _{i = 1}^n {\left|{{y_i} - \widehat{{y_i}}} \right|} \end{aligned}$$

where MSE represents the mean square error, also known as the L2 loss; MAE represents the mean absolute value error, also known as the L1 loss; n denotes the number of samples, $\widehat{{y_i}}$ represents the predicted value of the i-th sample, and ${{y_i}}$ represents the corresponding sample value.

The advantage of the MSE loss function is that its curve is continuous and differentiable, which is convenient for the calculation of the gradient descent algorithm. However, when the predicted value differs greatly from the true value, the square term will further increase the error. If there are outliers in the sample data, the model will become unstable. The MAE loss function can enhance the robustness to outliers, but its disadvantage is that the gradient is fixed and does not decrease as the error decreases. Therefore, MAE is often used in conjunction with a changing learning rate, and the learning rate is appropriately reduced when the error gradually decreases. In theory, the MAE loss function is more suitable for model training based on logging data.

For regression tasks such as logging curve prediction, the coefficient of determination (R-square) is often used to evaluate the prediction accuracy, which is defined as follows:

5 $$\begin{aligned} {R^2} = 1 - \frac{{\sum \nolimits _{i = 1}^n {{{\left( {{y_i} - {{{\widehat{y}}}_i}} \right) }^2}} }}{{\sum \nolimits _{i = 1}^n {{{\left( {{y_i} - {\overline{y}} } \right) }^2}} }} \end{aligned}$$

where $\widehat{{y_i}}$ represents the predicted value of the i-th sample, ${{y_i}}$ represents its corresponding sample value, and $\overline{y}$ represents the average of samples. R-Square can exactly describe the fitting degree between outputs and samples and the prediction accuracy of the model. Its maximum value is 1, and the closer to 1, the more accurate the prediction results are. If the R-square is negative, the prediction results are unreliable and need to be reconsidered. The R-square removes the influence of magnitude and dimension and takes 1 as the standard line, which is called the best indicator to measure the accuracy of linear regression^20^.

#### Weights updating

Based on the loss function, the process of updating network weights by the gradient descent algorithm is called backward propagation. According to the chain rule of the composite function, the partial derivative of the loss function to each neuron is calculated. The neuron subtracts the product of the partial derivative and learning rate to obtain the modified network weight. Forward and backward propagation repeat until the network converges.

Simple gradient descent algorithms, stochastic gradient descent (SGD) derives from a stochastic approximation method proposed by Robbins and Monro in 1951^21^ and was first applied to the perceptron proposed by Rosenblatt^22^. Its disadvantage is that the loss function descends slowly, which oscillates on both sides of the minimum. Based on SGD, momentum gradient optimization introduces the first-order momentum idea. When updating the weight, the direction of the last weight updating is considered to resolve the problem with falling into a local optimal solution. The Nesterov Acceleration Gradient (NAG)^23^ method first uses the historical gradient and then uses the current gradient to modify the gradient direction, which accelerates the weight updating algorithm. The adaptive gradient algorithm (AdaGrad)^24^ uses the number of iterations and the cumulative gradient to automatically attenuate the learning rate. The root mean square prop (RMSprop) algorithm is an unpublished, adaptive learning rate method proposed by Geoff Hinton in his Coursera Class. It adds an attenuation factor to the gradient accumulation based on AdaGrad to solve the problem that some iterative gradients are too large to change the adaptive gradient. The adaptive momentum estimation (Adam)^25^ optimizer combines the idea of a momentum gradient optimization and an adaptive learning rate, which basically solves a series of gradient descent problems and is a commonly used weight updating algorithm.

### Framework design of the prediction model

**Figure 4:**
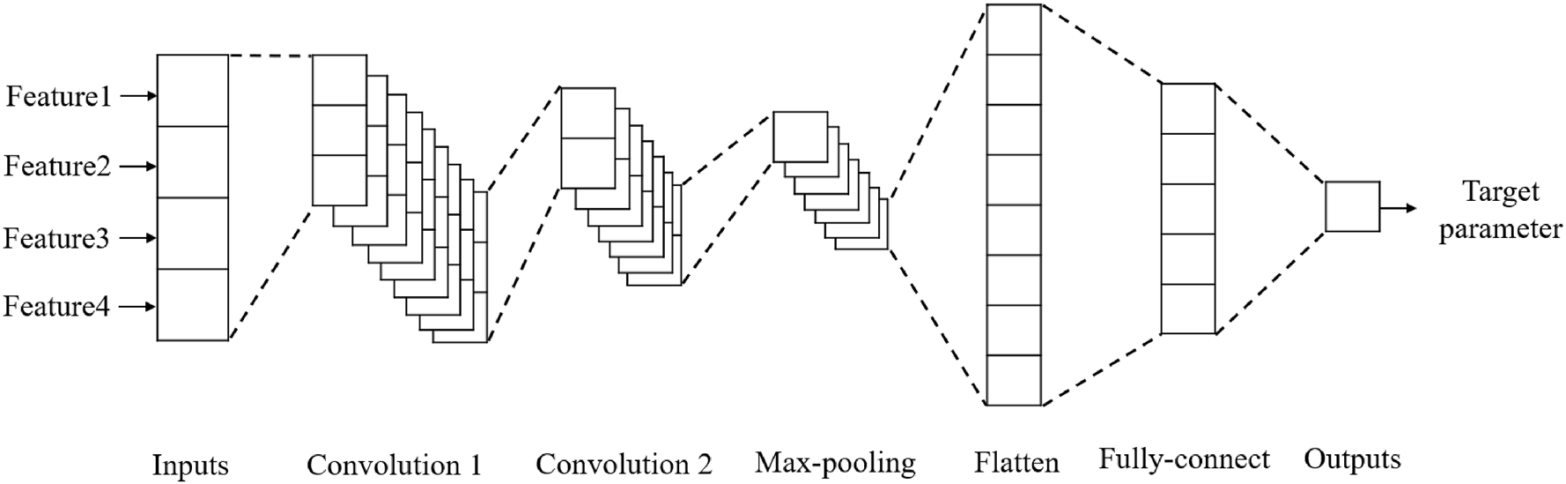
1D-CNN framework design.

Based on the study of 1D-CNN, a network structure is designed to implement the prediction of “sweet spot” parameters. Figure 4 shows a network structure with four logging parameters as input and a corresponding output. For each target “sweet spot” parameter, a network needs to be trained.

The network input is a vector of length 4 and passes through two one-dimensional convolutional layers. The first convolutional layer contains 128 filters of size 2 with ReLU activation function. And the second convolutional layer contains 64 filters of length 2 with ReLU activation function to obtain 64 high-level feature maps of length 2. After the maximum pooling layer, the feature length is reduced by half. Then flatten the feature maps and get a vector of length 32. The extracted features pass through a fully connected layer which contains 30 units, which performs a complex mapping between features and target parameter. Finally, the output layer corresponds to TOC, PHI, GAS or other target parameters. All of the one-dimensional convolutional layers and fully connected layers use batch normalization to improve the network performance. The output layer uses the sigmoid activation function to make the output value between (0, 1) for denormalization.

### Prediction workflow

The procedure of “sweet spot” parameter prediction is summarized as data preprocessing, model training and three-dimensional prediction.

Preprocessing includes data cleaning, sensitivity feature analysis, data normalization, etc. The preprocessed logging data is divided into a training set and validation set for model training and validation. The data of the validation set does not participate in the model training, and vice versa. Dropout and early stopping are appropriately used in model training to prevent overfitting. Hyperparameters of the network are adjusted according to the decline of the loss function and the prediction accuracy to obtain the optimal model. The training set and the validation set are redivided to test the stability of the network, and then the models with good performance are saved.

**Figure 5:**
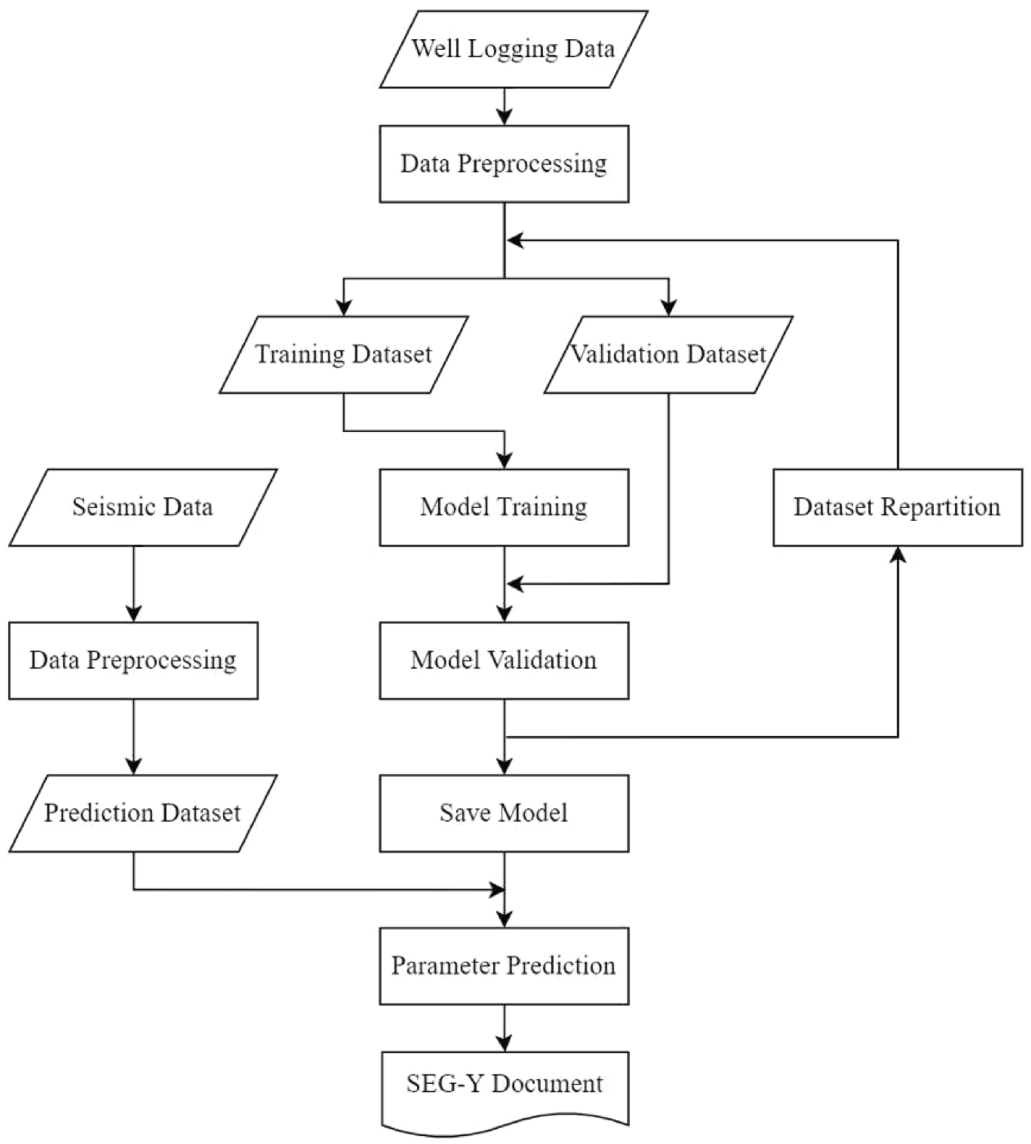
Prediction workflow.

Preprocessed seismic data is input into the model to predict the reservoir “sweet spot” parameter and generate a three-dimensional data to investigate the spatial distribution of the “sweet spot”. The process is summarized in Fig. 5.

## Applications

The Weirong shale gas field is located in the syncline structure of Baima Town, southeast of the Weiyuan structure in Sichuan Basin. The target reservoir is called Ordovician Wufeng–Silurian Longmaxi Formation, which is a deeply buried, abnormally high-pressure continuous shale gas reservoir with a burial depth of 3550–3880 m and a formation pressure coefficient of 1.94–2.06. The Wufeng–Longmaxi Formation shale reservoir is of good quality, 25–39 m thickness, with high TOC and GAS and has great exploitation potential^26^. However, the problems of low “sweet spot” evaluation accuracy and low single-well production urgently need to be improved. Therefore, based on deep learning, high-precision intelligent parameter prediction method is of great importance to the optimization of the “sweet spot” target of shale reservoirs. The current work of the study area is aimed at the evaluation of geological “sweet spot”, and therefore, TOC, PHI and GAS are taken as examples to analyze the effect of the prediction method. However, prediction models with strong generalization ability and stable performance can be generalized to the prediction of other important “sweet spot” parameters such as permeability, pressure condition and brittleness index.

### Sensitive parameter analysis

The input of the prediction model is determined according to the sensitive parameter analysis of the GAS, TOC and PHI in the study area. Well logging WY29 is used as an example to demonstrate the analysis of sensitive parameters.

**Figure 6:**
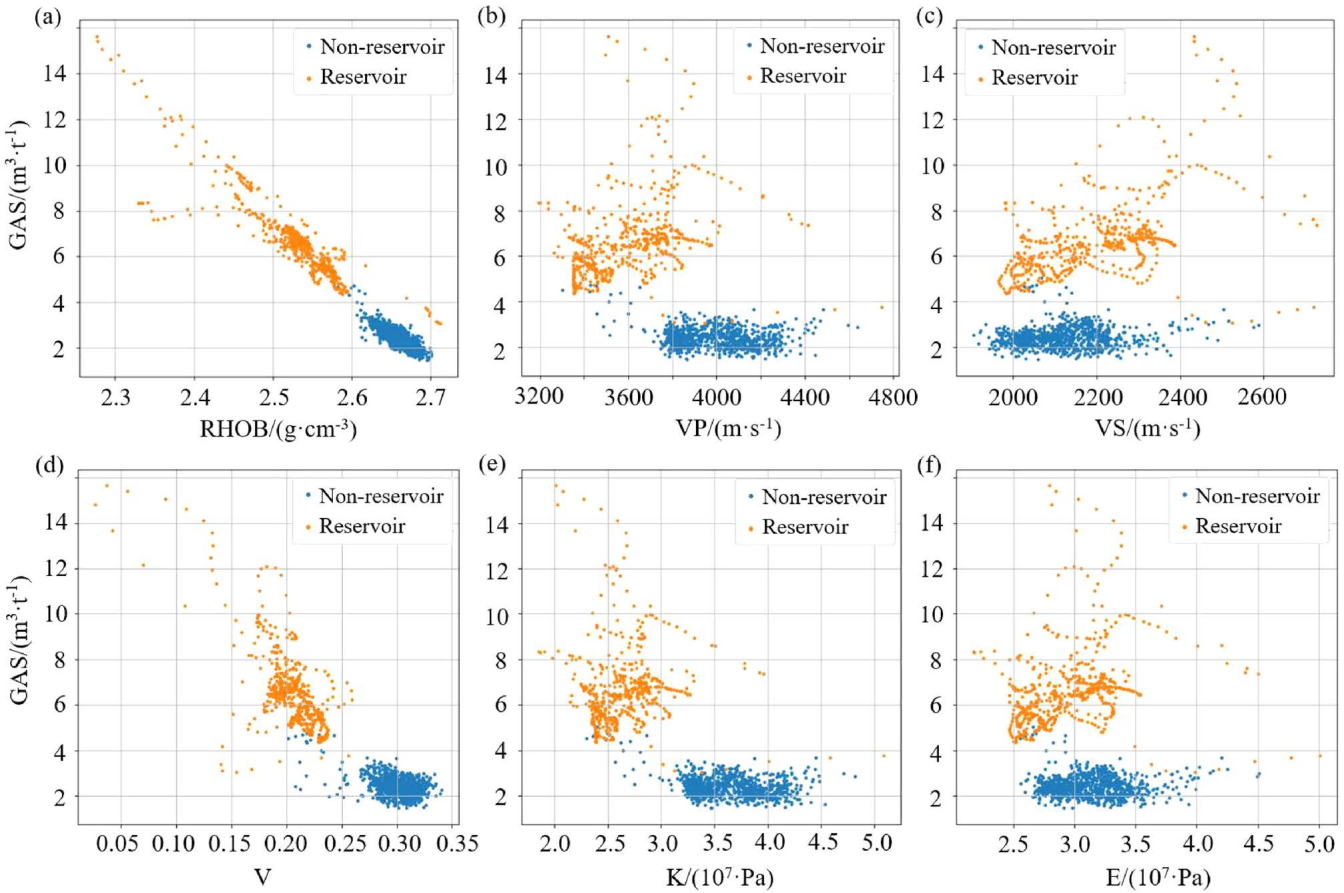
Cross plot of GAS with (**a**) density, (**b**) longitudinal wave velocity, (**c**) transverse wave velocity, (**d**) Poisson’s ratio, (**e**) bulk modulus and (**f**) Young’s modulus.

Cross plot recognition technology is widely used in oil and gas exploration, and it plays an important role in checking the quality of logging data, selecting sensitive parameters, determining lithology and testing interpretation results^27^. Tian et al provides a new approach to identifcation and quantifcation gas hydrate in unconsolidated marine sediments based on velocity cross plot analysis^28^. The cross plots of GAS with some logging parameters are shown in Fig. 6. According to the known shale gas reservoir depth, WY29 logging data is divided into reservoir and non-reservoir data. The reservoir has high GAS values.

Figure 6a shows that there is a negative correlation between GAS and density (RHOB), where the higher GAS value corresponds to the lower density. In other words, reservoirs and non-reservoirs with difference GAS values can be distinguished by RHOB. Although there is a certain degree of linear correlation between gas and RHOB, it can be seen from the cross plot that the the sample points are distributed around the regression line and there is some interference from the wild values. For such problems, the nonlinear models has stronger noise immunity and generalization ability than linear regression or linear neural networks^29^. In Fig. 6b, GAS has no obvious linear relationship with longitudinal wave velocity (VP), but reservoir and non-reservoir points can be divided. Reservoir points with high GAS values are distributed in the VP range of 3300–3900 m/s, and non-reservoir points with low GAS values are concentrated at the VP of 3750–4300 m/s. Figure 6c shows that there is no obvious distribution rule between GAS and transverse wave velocity, but there may be some complex and hidden feature information.

The existing parameters of the well logging are used to calculate the rock elastic mechanics parameters such as Poisson’s ratio, bulk modulus, and Young’s modulus and to analyze the feature mapping relationship with GAS^30^. The calculation is as follows:

6 $$\begin{aligned} V= & {} \frac{{0.5{{\left( {Vp/Vs} \right) }^2} - 1}}{{{{\left( {Vp/Vs} \right) }^2} - 1}} \end{aligned}$$

7 $$\begin{aligned} K= & {} \rho \cdot V{p^2} - \frac{4}{3}V{s^2} \end{aligned}$$

8 $$\begin{aligned} E= & {} \frac{{\rho \cdot V{s^2} \cdot \left( {3V{p^2} - 4V{s^2}} \right) }}{{V{p^2} - V{s^2}}} \end{aligned}$$

where V, K and E represent Poisson’s ratio, bulk modulus, and Young’s modulus; $\rho$, Vp and Vs represent the density, longitudinal wave velocity and transverse wave velocity. Cross plots of GAS with the above three rock elastic mechanics parameters are shown in Fig. 6d–f.

There is a certain degree of negative correlation between GAS and Poisson’s ratio. Higher GAS corresponds to a lower Poisson’s ratio, and the distinction between reservoirs and non-reservoirs is obvious in Fig. 6d. In Fig. 6e, the cross plot of GAS with the bulk modulus shows good separability between reservoirs and non-reservoirs. There is no obvious distribution characteristic between GAS and Young’s modulus, as shown in Fig. 6f. Thus, Young’s modulus is not used as the input feature of the network.

In summary, density, longitudinal wave velocity, Poisson’s ratio and bulk modulus are the preferred input features for GAS prediction. In the sensitive feature analysis of TOC and PHI, the negative correlation with density is stronger, and the longitudinal wave velocity, Poisson’s ratio and bulk modulus show good separability for reservoirs and non-reservoirs. Therefore, the above four parameters can be used to predict GAS, TOC, and PHI. Each depth-domain sample point of the logging data corresponds to a group of training data, which contains an input feature of length 4 and a target parameter.

The rock elastic mechanics parameters are calculated using definite formulas. Although the CNN has powerful feature extraction capabilities, it is automated and unexplainable. Therefore, the use of appropriate preprocessing method on the input data is beneficial to the network training.

### Model training and validation with well logging data

#### Data standardization

Data preprocessing is of great importance in deep learning. The sample data is properly preprocessed to ensure that the model training is carried out correctly and to improve the prediction performance of the model.

**Figure 7:**
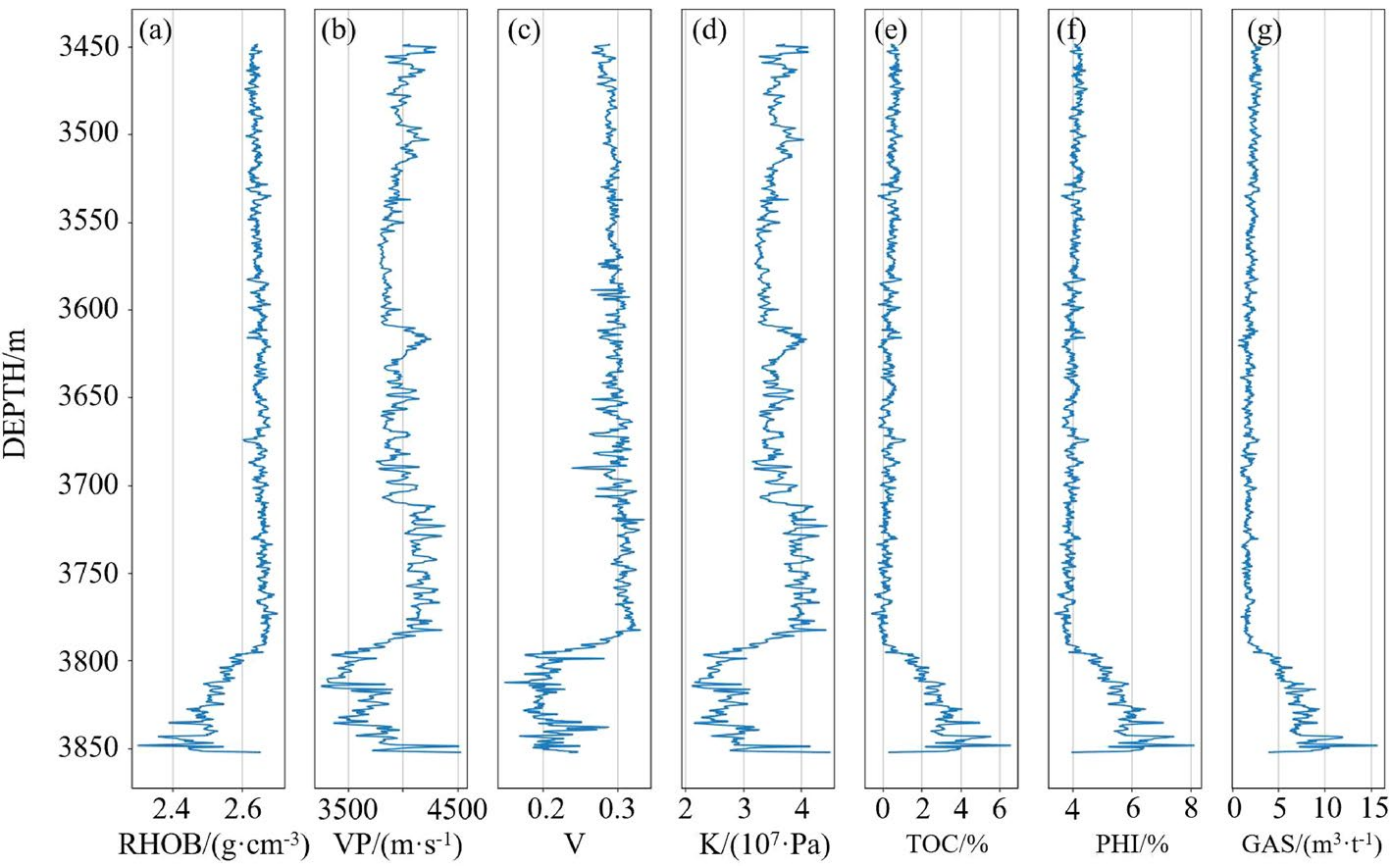
Well logging curves of WY23. (**a**) density, (**b**) longitudinal wave velocity, (**c**) Poisson’s ratio, (**d**) bulk modulus, (**e**) total organic carbon content, (**f**) porosity and (**g**) gas content.

First, the invalid logging data and outliers are removed. According to the depth-time correspondence between logging and seismic data, logging data with the same stratum range as the seismic data is selected to ensure the consistency of the stratum. Formulas (6) and (7) are used to calculate logging Poisson’s ratio and bulk modulus, which together with density and longitudinal wave velocity form the input features. Figure 7 shows the WY23 logging curve, which includes four input features and three target parameters. The data of target reservoir (3790–3850 m) with high TOC, GAS and PHI take a small proportion of the sample data, and thus, it appropriately reduces non-reservoir data to ensure the balance of sample data.

The difference in the numerical magnitude of the sample data causes numerical problems in the network weights, making the model unable to converge. Therefore, the dataset is normalized to remove the dimensional influence and unify the magnitude. Commonly used transformation methods include standardization and maximum normalization, which are calculated as follows:

9 $$\begin{aligned} {x_{std}}= & {} \frac{{x - {\overline{x}} }}{\sigma } \end{aligned}$$

10 $$\begin{aligned} {x_{scaled}}= & {} \frac{{x - {x_{\min }}}}{{{x_{\max }} - {x_{\min }}}} \end{aligned}$$

where ${x_{std}}$ represents the standardized value, ${x_{scaled}}$ represents the maximum normalized value, and *x*, ${{\overline{x}} }$, $\sigma$, ${x_{\min }}$ and ${x_{\max }}$ are respectively the original value, sample mean, sample standard deviation, minimum and maximum. Standardization makes the sample data obey a normal distribution with a mean of 0 and a standard deviation of 1. And the maximum normalization maps the value of sample data to the range of (0, 1).

In the experiment, it was found that purely using standardization or maximum normalization to process the sample data cannot obtain an ideal training result.

The data range of various wells is quite different. For example, the GAS range of well WY23 is (0.67, 15.68), and in well WY11, it is (0, 5.54). When using the same normalization model, the GAS value of well WY23 is mapped to (0.05, 1), and WY11’s GAS value is mapped to (0, 0.36), which are unbalanced. Values with large range differences make different contributions to weight updating in network training. Thus, it is inappropriate to normalize data with the same model. Fitting a separate normalization model for each well loses the comparability between wells and leads to numerical confusion.

Then, data standardization is considered. The data of each well is standardized, making it obeys the standard normal distribution, which can preserve the comparability of wells. However, there are still large differences in the numerical range of the standardized well data. For example, the standardized density of WY23 is in the range of (− 6, 1.3), and the GAS is in the range of (− 1, 6.7), which is not conducive to the update of neural network weights. Therefore, all of the standardized well logs are normalized with the same maximum normalization model, enabling the data range from 0 to 1. It is necessary to save the normalization and standardization parameters so that the validation data can be normalized and standardized. The parameters will also used for the denormalization and destandardization of the validation outputs. Because of the standardization, the validation inputs has the similar distribution with the training inputs, then after normalization, the range of the inputs tends to be similar. In addition, the sigmoid function in the output layer has a constraint effect, so the outputs can be reverted to the correct range.

Processed well logging data is suitable for the training and validation of neural networks. And data of different wells have similar statistical features, which can be assumed to be ergodicity^31^. The processing parameters of the training set can be used to validation set.

#### Model training and validation

**Figure 8:**
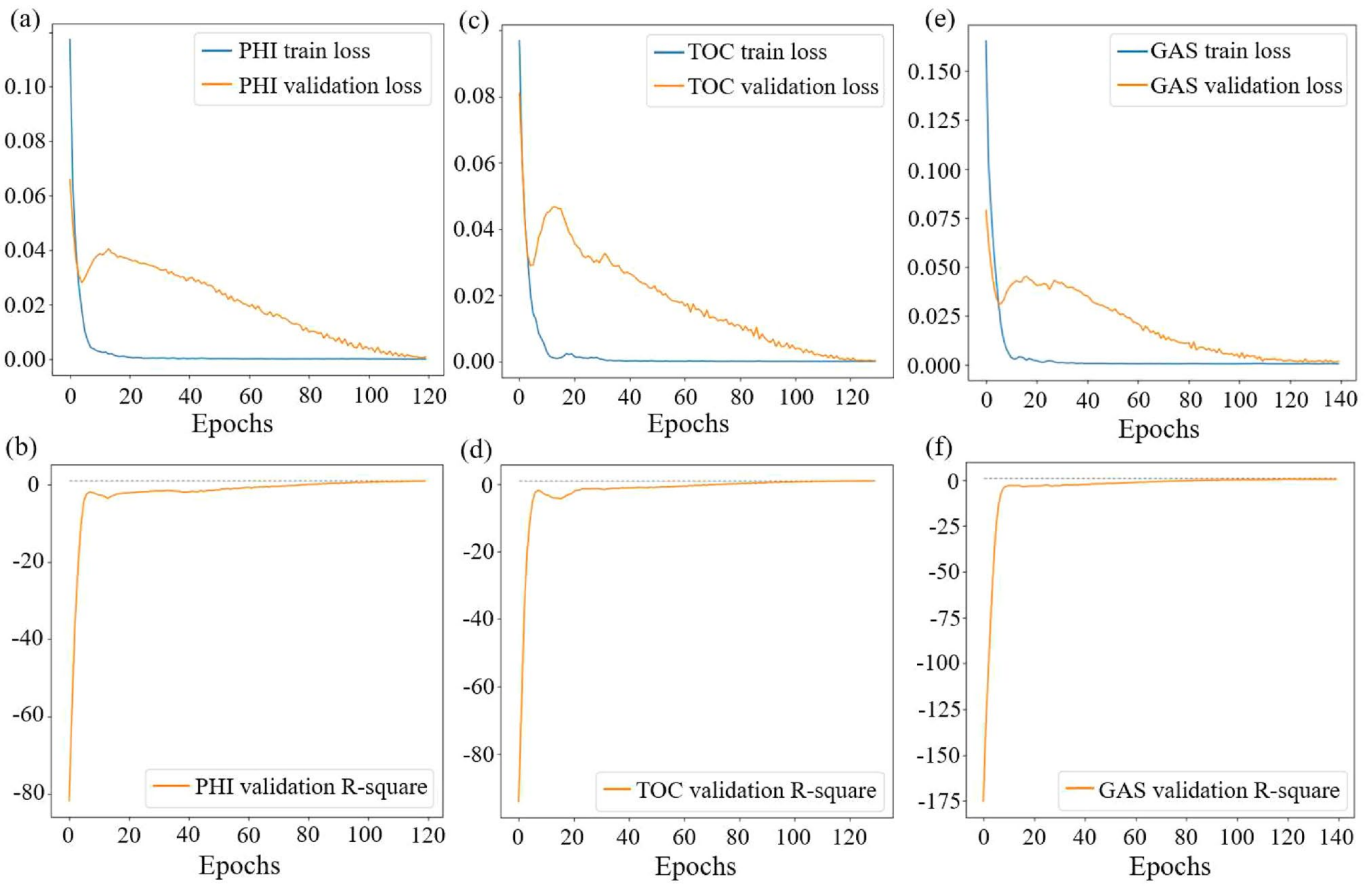
Training curves of PHI, TOC and GAS predict model.

The preprocessed well logging data is divided into the train set and validation set. The train set includes data from well WY29 and WY35, which is used to train the model until convergence, and the validation set , includes data from well WY23 and WY11, verifies the accuracy and stability of the model. Figure 8 shows the loss and R-square curves during the training of the PHI, TOC and GAS prediction models.

Figure 8a,c,e show that each train loss drops quickly in the first 10 epochs and plateaus after 40 epochs in each model. The validation loss is reduced to a low level after 120 epochs for the model to converge. The validation R-square approaches one after 100 epochs in Fig. 8b,d,f, which means that the accuracy of the model has reached a high level.

In the experiment, it was found that the well logging data preprocessed by separate standardization and then maximum normalization are more reliable, with which the model was trained to have the best prediction result.

**Figure 9:**
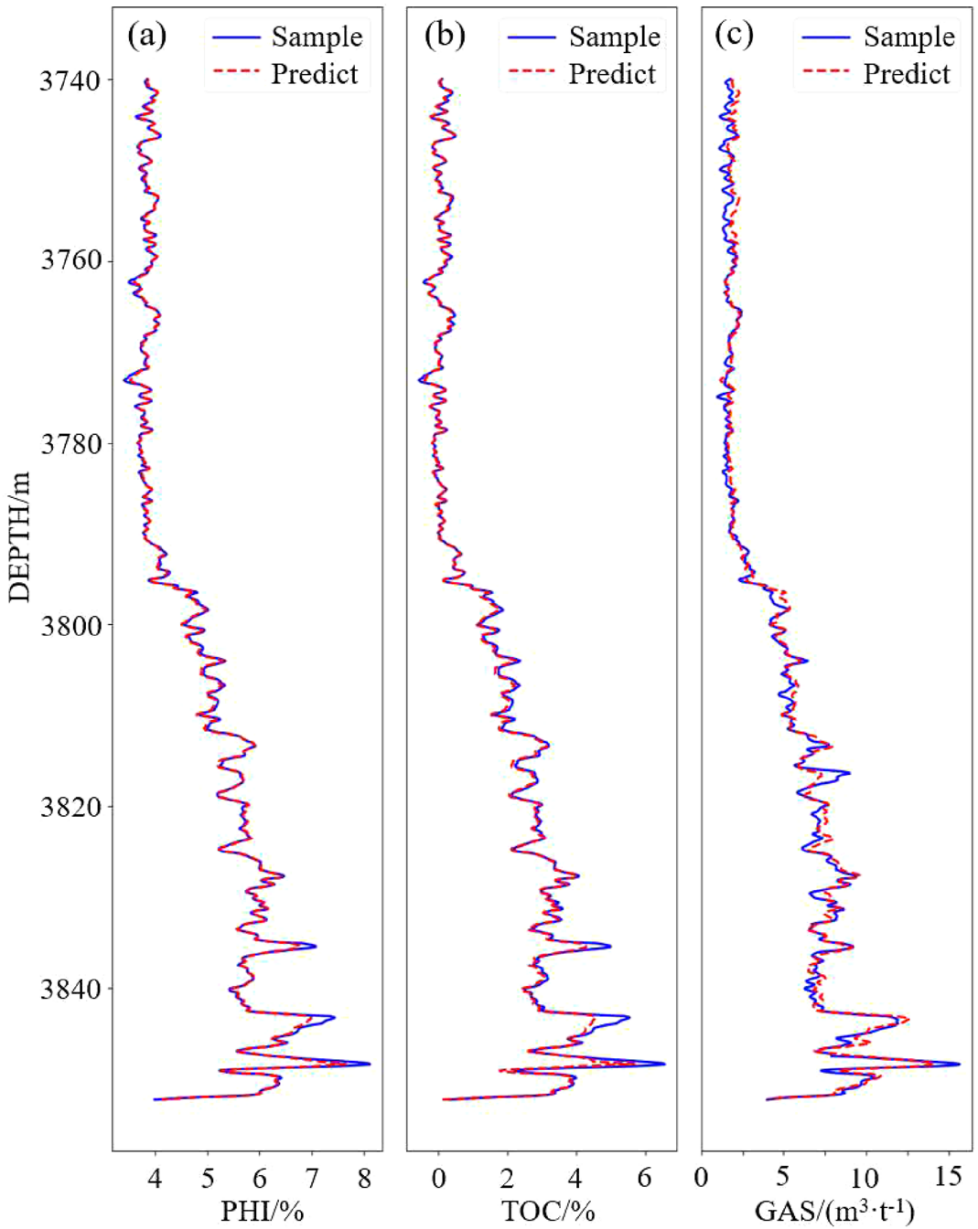
Comparison of predictions and samples of well WY23.

**Figure 10:**
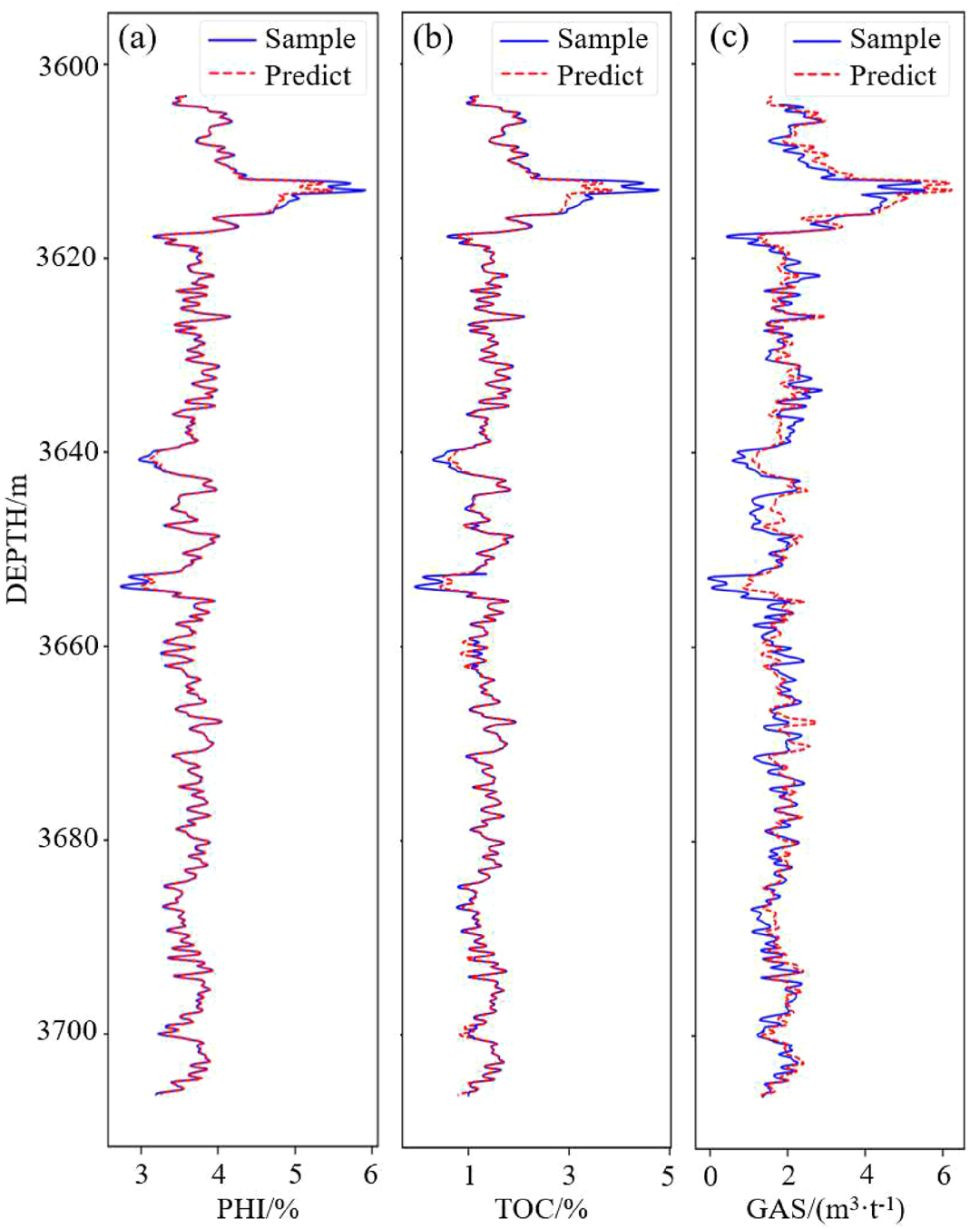
Comparison of predictions and samples of well WY11.

The comparisons between predicted values and sample values are shown in Fig. 9. The PHI and TOC prediction results fit the sample values very well, and the R-square reaches 0.99. The parameter GAS is affected by more complicated factors and has a stronger nonlinear relationship with density, meaning that it is more difficult to predict than TOC and PHI, which is the reason why the training of the GAS prediction model needs more epochs to converge. The GAS prediction accuracy is slightly lower than TOC and PHI, but it can still reach 97%. Therefore, the 1D-CNN model can accurately predict the logging TOC, PHI, and GAS curves based on density, longitudinal wave velocity, Poisson’s ratio and bulk modulus. Figure 10 shows the validation curves from well WY11. It can be seen that the predicted TOC and PHI have a high fitting degree with the samples, witch is higher than 0.98. The accuracy of GAS prediction is also lower than that of the TOC and PHI. The main reason why the validation accuracy of well WY11 is lower than WY23 is that the WY11 located far away from the well WY23, WY29 and WY35, so that the features of the data are slightly different. Despite this, the model is still able to predict the parameters with high accuracy.

For comparison, different machine learning methods, such as linear regression (LR), support vector regression (SVR), Multilayer Perceptron (MLP) and K-nearest neighbor regression (KNN), are applied to the same training and validation dataset^32^. LR are suitable for linearly distributed data and has scarcely parameters to be adjusted. MLP, in principle, is the stack and connection of linear units with nonlinear activation function. The MLP model used in the experiment has 3 hidden layers, and respectively contains 128, 64, 32 units with ReLU function. The output layer has one unit with sigmoid function. The experiment process is similar to that of the 1D-CNN, and the model costs 120 epochs to convergence. SVR is an application of support vector machine (SVM) to regression problems. The important parameters include penalty coefficient (C), kernel function and kernel function coefficient (gamma). Grid search method is applied to optimize the hyperparameter tuning and the specific settings are as follows. The grid used four kernels, linear, radial basis function (RBF), sigmoid and polynomial. The penalty coefficient has three options, 0.1, 1 and 10. Gamma has five options, 0.001, 0.01, 0.1, 1 and 10, and note that it is invalid for linear kernel. Training and cross-validation show that the optimal combination of hyperparameters is RBF kernel with gamma = 0.1 and C = 1. KNN regression is also used in conjunction with grid search method to get the optimal hyperparameters. The neighbors number (K) has five options, 3, 5, 7, 9 and 11. The neighbors weight can set to uniform or depend on the distance, and for distance, manhattan distance, euclidean distance and chebyshev distance are candidates. After experimentation, the optimal hyperparameters is K=9 and euclidean distance as weight. The average MSE, MAE and R-square between predictions and samples of each method is shown in Table 1.

**Table 1:** Prediction accuracy of 1D-CNN, MLP, SVR, KNN and LR method.

Parameter	Metrics	1D-CNN	MLP	SVR	KNN	LR
PHI	MSE	0.0000395	0.0000812	0.000332	0.0000682	0.0000560
	MAE	0.00312	0.00656	0.0139	0.00413	0.00723
	R-Square	0.994	0.989	0.956	0.991	0.983
TOC	MSE	0.0000679	0.000177	0.000282	0.0000952	0.000267
	MAE	0.00729	0.0115	0.0128	0.00983	0.0121
	R-Square	0.991	0.975	0.961	0.987	0.985
GAS	MSE	0.000201	0.000299	0.000265	0.000374	0.000859
	MAE	0.0104	0.0128	0.0124	0.0137	0.0232
	R-Square	0.974	0.962	0.966	0.953	0.948

#### Parameter prediction with seismic data

On the basis of prediction model training and validation, parameter TOC, PHI and GAS of the study area are predicted based on seismic data to generate three-dimensional data. Then, the spatial distribution of “sweet spot” parameters can be investigated. The preprocessing of seismic data is the same as the well logging data. For each seismic trace, the null values are removed, and Poisson’s ratio and bulk modulus are calculated according to Formula 6 and 7. The input features are first standardized and then normalized to obtain the feature set.

The feature set is input into the 1D-CNN model for prediction, and the output values of the “sweet spot” parameter model are obtained. The outputs need Firstly destandardization and then denormalization to obtain the single-trace “sweet spot” parameter prediction result. The prediction results are written into the corresponding position of the SEG-Y data. The above operations are performed on all seismic traces, and the 3D seismic data is obtained. Professional seismic data visualization software is used to investigate the prediction effect.

**Figure 11:**
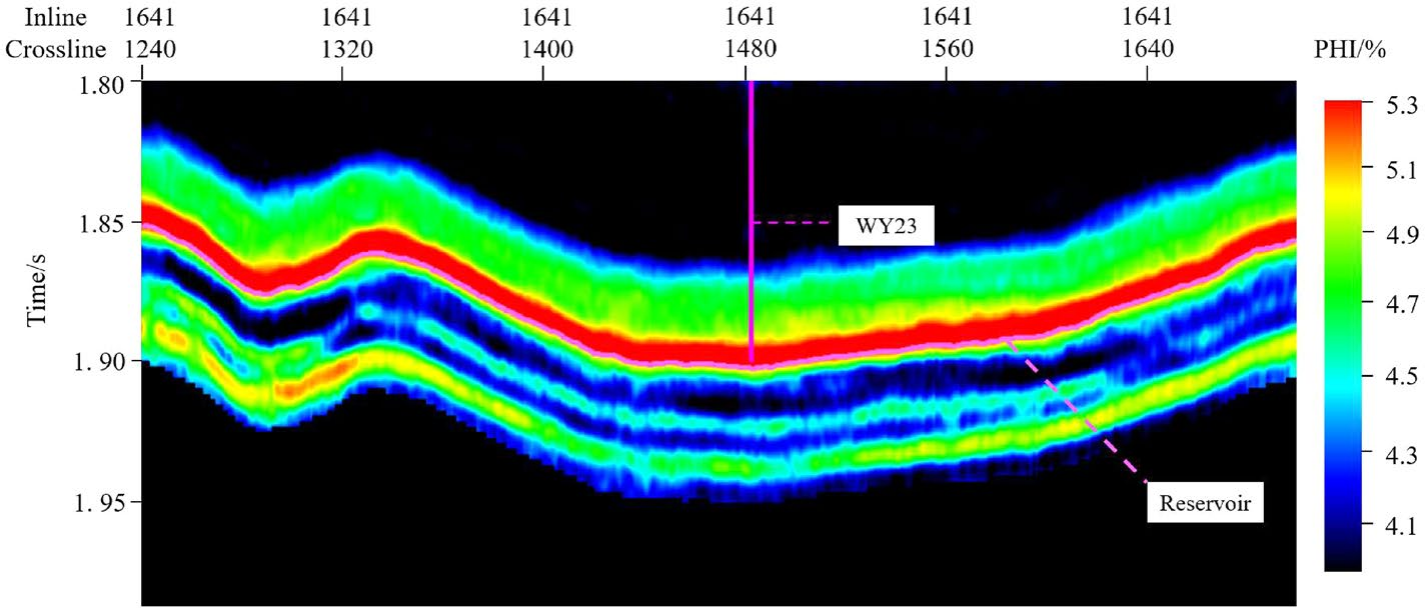
PHI section view of the Longmaxi formation.

**Figure 12:**
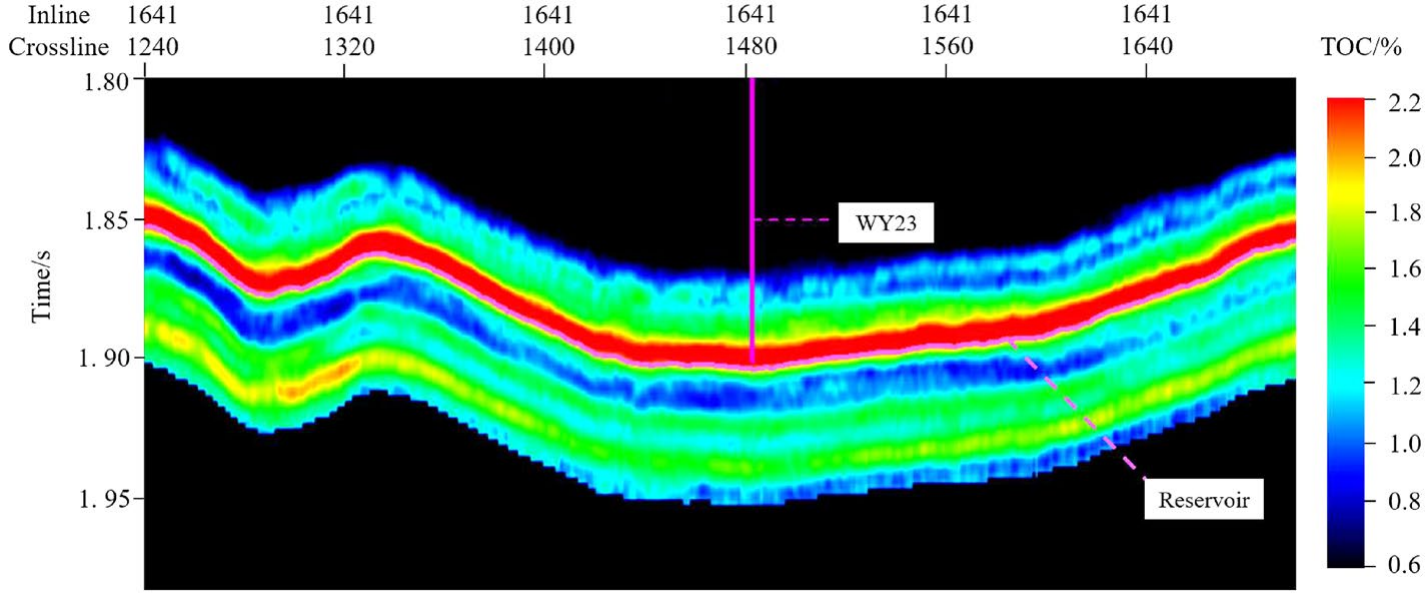
TOC section view of the Longmaxi formation.

**Figure 13:**
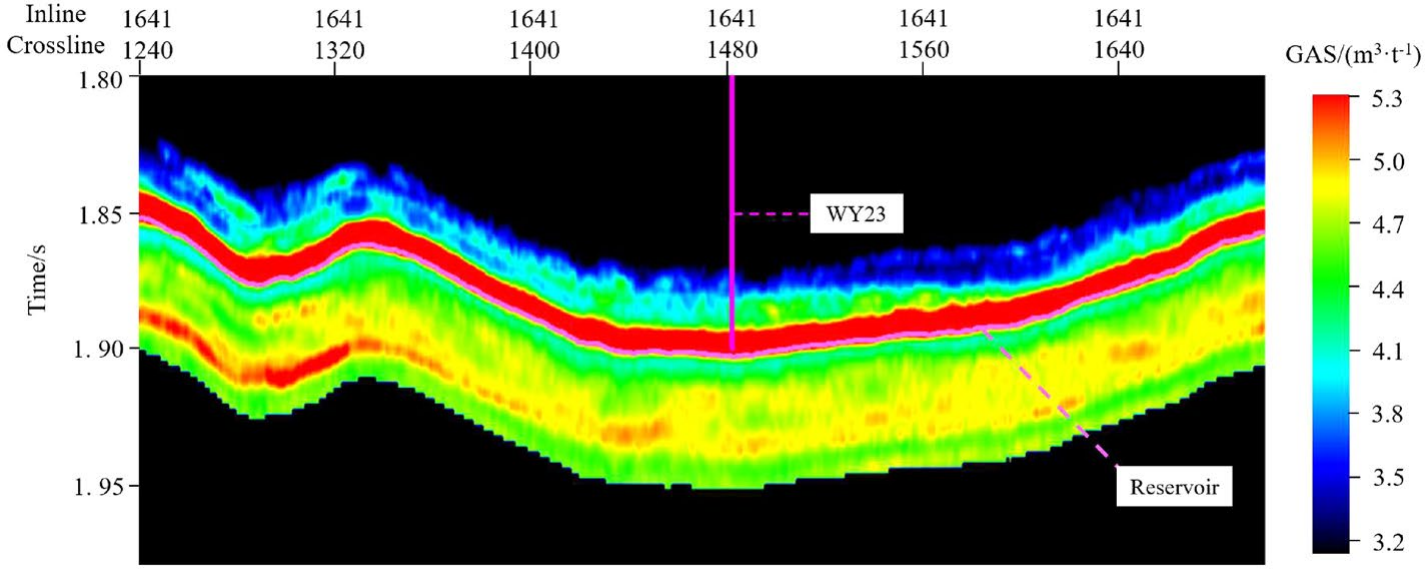
GAS section view of the Longmaxi formation.

Figures 11, 12 and 13 show the parameter PHI, TOC and GAS prediction results section view around well WY23. The target reservoir layer is the Wufeng-Longmaxi Formation. In the section views, it can be seen that the TOC, PHI and GAS in the reservoir layer are relatively higher than those in non-reservoir layer, with a thickness of approximately 20 m and good lateral continuity. The high TOC value reveals that the reservoir layer has good hydrocarbon generation conditions, and the high PHI value is beneficial to oil and gas storage, while also providing a good storage space for shale gas.

**Figure 14:**
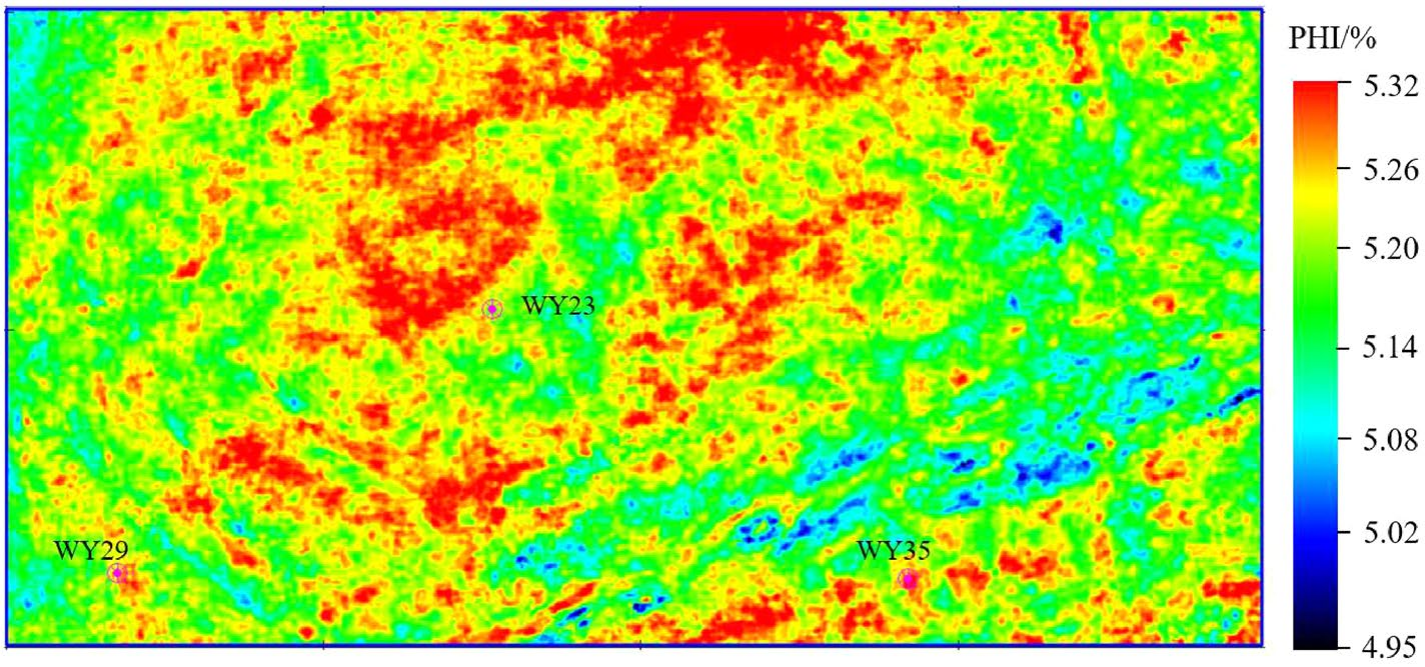
PHI distribution of the target reservoir layer.

**Figure 15:**
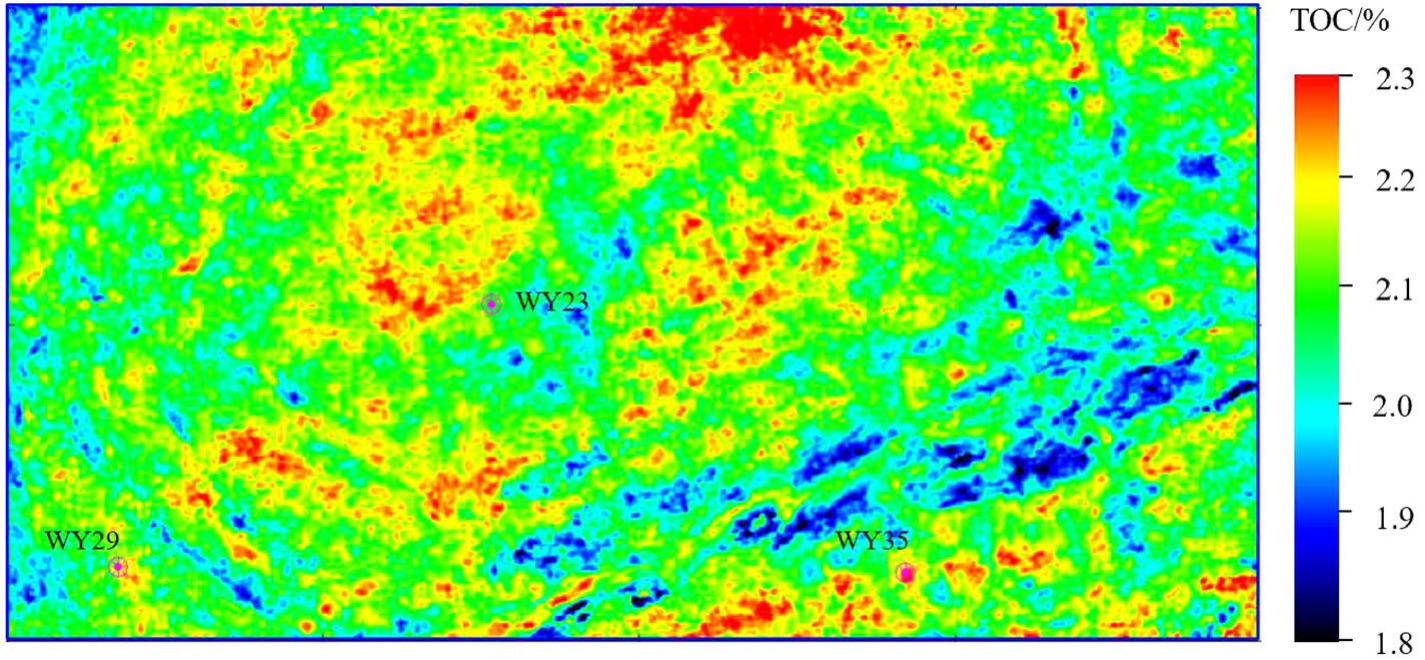
TOC distribution of the target reservoir layer.

**Figure 16:**
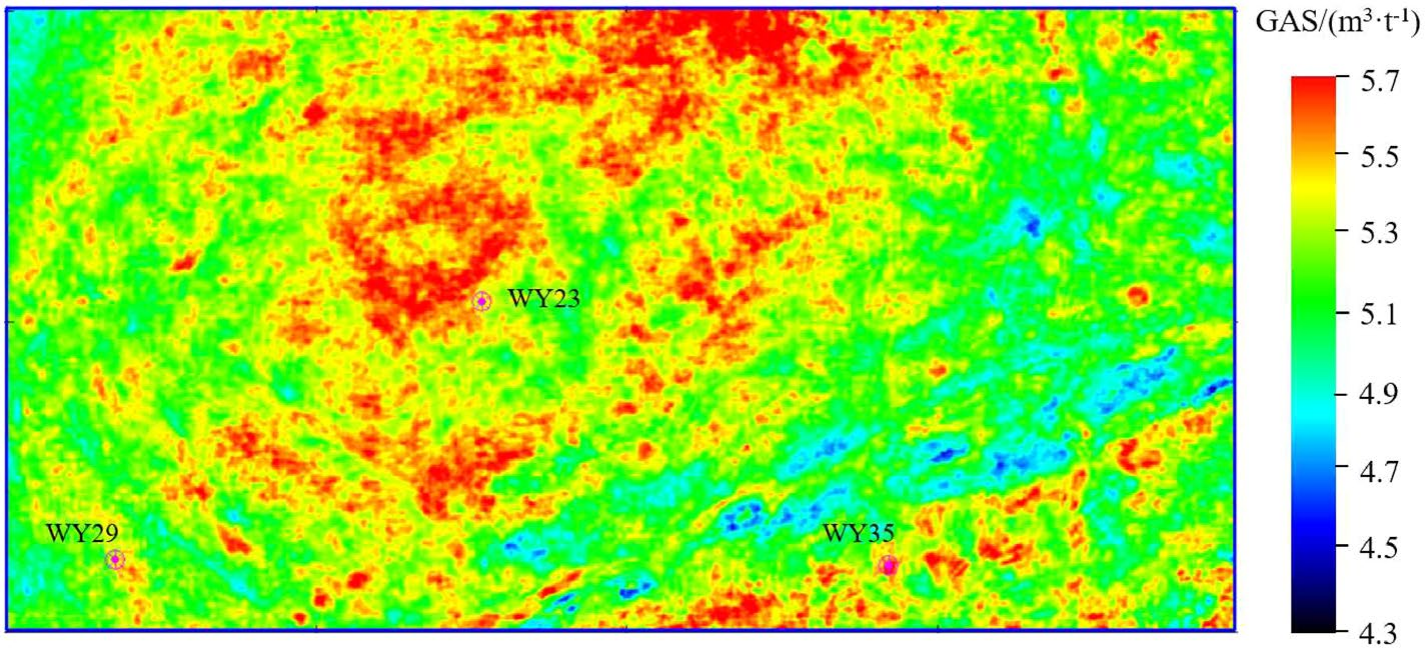
GAS distribution of the target reservoir layer.

Figures 14, 15 and 16 show the horizontal distribution of the sweet parameters of PHI, TOC and GAS along the target reservoir layer. In the three figures, the general distribution of GAS, TOC and PHI are similar, and the reason is that the well logging data used for model training has a high correlation between TOC, PHI and GAS. Around wells WY23, WY29 and WY35, GAS, TOC and PHI value are relatively high, showing the advantages of good hydrocarbon generation, strong storage capacity and a high gas content. The three wells achieve relatively high unimpeded shale gas flows of 280,000 m$^{3}$/day, 180,000 $^{3}$/day, and 230,000 $^{3}$/day. The predicted results are consistent with the actual drilling output of shale gas.

**Figure 17:**
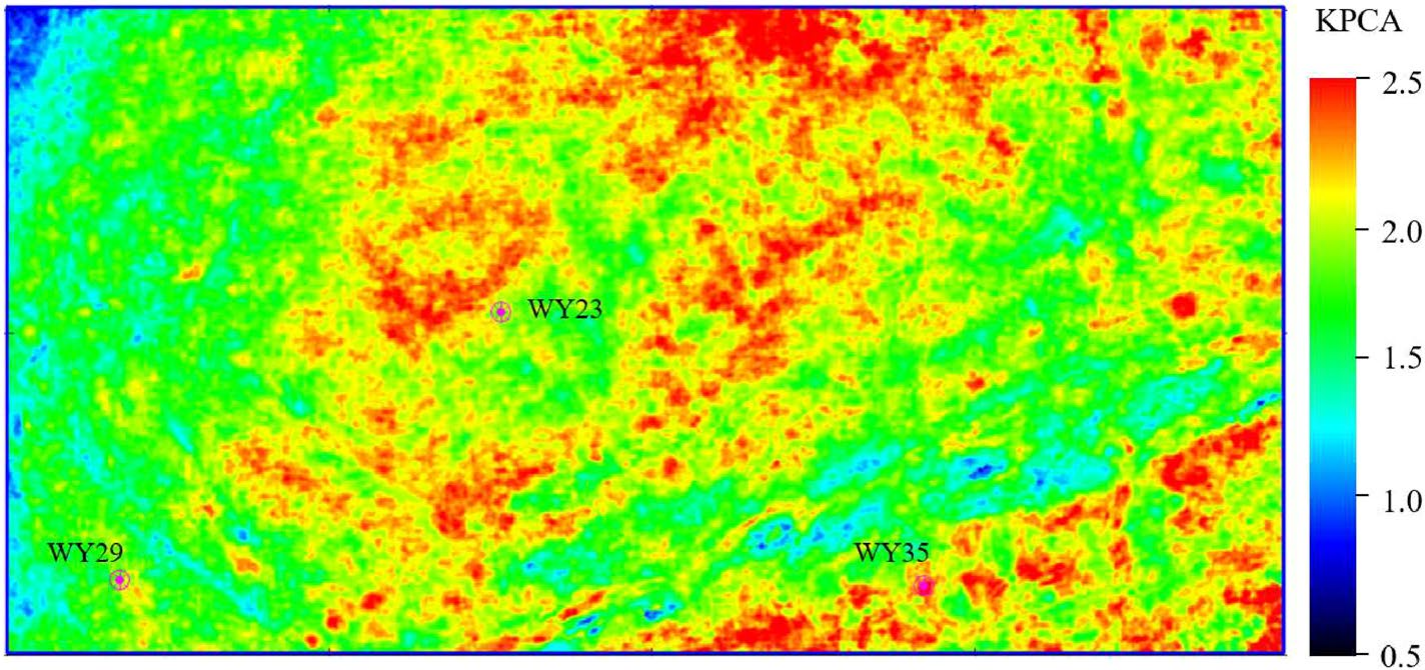
The first component of the KPCA result of the TOC, PHI and GAS.

Kernel principal component analysis (KPCA) was applied to obtain a comprehensive evaluation indicator from the three predicted parameters, and Fig. 17 shows the first component of the KPCA result. It can be seen that it has good similarity with each parameter, and can be used as a comprehensive evaluating indicator of shale gas geological “sweet spot”.

In summary, based on the 1D-CNN deep learning method, combined with reservoir “sweet” sensitive feature optimization, high-precision geological “sweet spot” parameter prediction can be realized. The spatial distribution of the geological “sweet spot” in the reservoir layer can be accurately predicted, and the well-seismic coincidence is good, which confirms the effectiveness of the method.

## Conclusion

- The geological “sweet spot” parameter prediction based on 1D-CNN can make full use of the advantages of convolutional neural networks. For supervised learning, sample data and preprocessing method play a key role in the performance of the model. Therefore, we first analyze the sensitive features of the geological “sweet spot” parameter to determine the model inputs. Due to the characteristics of well logging data, a method of single-well standardization and then unified maximum normalization is proposed to improve the fitness between the data and the model. Model optimization techniques, such as dropout, batch normalization and early stopping are used during model training to obtain the optimal model training state.
- The reservoir geological “sweet spot” parameter prediction model based on well logging data has high accuracy, strong stability and good generalizability. The R-square score of TOC and PHI prediction can reach 99%, and the GAS prediction R-square reaches 97%. Compared with the BP network, SVR and KNN methods, the 1D-CNN has the highest prediction accuracy. The model can predict a variety of parameters and can be used to predict the geological “sweet spot” parameters of shale reservoirs with seismic data.
- Based on the prestack inversion seismic data of the Weirong shale gas field, combined with the geological “sweet spot” parameter model, the spatial distribution of the parameters can be precisely predicted. In areas with higher TOC, PHI and GAS, high industrial shale gas has been obtained, which means that the predicted result matches the actual distribution. The convolutional neural network-based shale reservoir geological “sweet spot” parameter prediction method is reliable and can provide method support for the optimization of shale gas exploration targets and the improvement of single well gas production.

## Data Availability

The data that support the findings of this study are available from the corresponding author upon reasonable request.
